# Supplementary material for: Assessment of health system readiness for routine maternal and newborn health services in Nepal: Analysis of a nationally representative health facility survey, 2015
Source: PLOS Glob Public Health. 2022 Nov 21;2(11):e0001298. doi: 10.1371/journal.pgph.0001298 (PMC10022376; doi:10.1371/journal.pgph.0001298)
Supplement: S1 File — Table A: Service availability and readiness assessment items for ANC services. Table B: Service availability and readiness assessment items for perinatal services. (DOC) [file pgph.0001298.s001.doc]

**S1 File: Services availability and facility readiness items for MNH services in Nepal**

**Table A: Service availability and readiness assessment items for ANC services**

| **Service availability domain:** |
| --- |
| **Services availability items** |
| ANC counselling |
| Birth preparedness package counselling |
| Albendazole tablets distribution |
| Newborn care counselling |
| Family Planning counselling |
| Breastfeeding counselling |
| PNC counselling |
| Tetanus toxoid service |
| BP Measure service |
| Weighting clients |
| HIV prevention counselling |
| Iron tablet distribution |
| Folic acid distribution |
| HIV test and counselling |
| Measure height |
| Health education service |
| Misoprostol distribution |
| Anaemia test service |
| Urine protein test |
| Urine test service |
| Haemoglobin test services |
| **Facility readiness domain** |
| **General readiness** |
| Client latrine |
| Client waiting area |
| Water supply |
| Electricity service |
| Emergency transport |
| Landline phone |
| 24-hour staff availability |
| **Medicine** |
| Albendazole tablets |
| Tetanus toxoid vaccine |
| Iron-folic tabs |
| Folic acid |
| Misoprostol tablets |
| **Equipment** |
| Examination table |
| Autoclave service |
| Fetoscope available |
| Weighing scale |
| Stethoscope |
| BP set manual |
| Thermometer |
| Disinfectant for IP |
| Soap for Infection Prevention (IP) |
| Water for infection prevention |
| Examination light |
| Tape fundal height |
| Digital blood pressure (BP) tool |
| **Staff training and guidelines** |
| Supervision of staff |
| IEC materials for ANC service |
| ANC guideline |
| Complication and management |
| ANC counselling training |
| ANC screening training |
| Nutritional assessment |
| Other training (e.g., refresher training on ANC) |

Table B: Service availability and readiness assessment items for perinatal services

| **Service availability domain** |
| --- |
| **Newborn care services** |
| Immediate breastfeeding |
| Wrapping baby |
| Weighing newborn |
| Head to toe examination |
| Kangaroo mother care |
| Skin to skin contact |
| Delayed bathing |
| Use of chlorhexidine |
| Newborn resuscitation |
| Injectable antibiotic available |
| **Delivery care services** |
| Oxytocin parental |
| Use of paratograph |
| Injectable antibiotic available |
| Antibiotics parental |
| Anticonvulsant parental |
| **Facility readiness domain** |
| **General readiness** |
| Client latrine |
| Protected client waiting area |
| Water supply |
| Electricity service |
| Emergency transport |
| 24-hour duty call |
| Landline phone |
| Mobile phone |
| **Medicines** |
| Betadine solution |
| Intravenous fluid |
| Tablet oxytocin |
| Tablet magnesium sulphate |
| Chlorhexidine tube |
| Injectable antibiotics |
| Calcium gluconate |
| Nifedipine capsule |
| **Equipment** |
| Autoclave services |
| Delivery bed |
| Fetescope |
| Latex gloves |
| Infant scale |
| Sponge holder |
| Stethoscope |
| Delivery set |
| Cord cutting blade |
| Needle holder |
| Suturing blade |
| Bag and Mask |
| Epitomy set |
| Blood pressure set |
| Forceps |
| Disinfectant |
| Blank paratograph |
| Baby wrappers four sets |
| Thermometer |
| Vaginal speculum |
| Soap available in the maternity room |
| Cord clamper |
| Water available in the delivery room |
| Nayano Jhola set |
| Examination light |
| Dee-lee suction |
| Alcohol for hand rub |
| **Staff trainings and guidelines** |
| Supervision of health workers |
| External supervision in the last four months |
| Exclusive breastfeeding training |
| Neonatal resuscitation training |
| Kangaroo Mother Care training |
| Reproductive health guideline |
| Cord cutting training |
| Integrated management of pregnancy and childbirth |
| Acute management of the third stage of labour |
| Thermal care |
| Routine Labour and delivery |
| Maternal and newborn care update emergency obstetric care |
| Neonatal sepsis management |
| Other training (e.g., refresher training) |
