# Supplementary material for: Assessment of health system readiness for routine maternal and newborn health services in Nepal: Analysis of a nationally representative health facility survey, 2015
Source: PLOS Glob Public Health. 2022 Nov 21;2(11):e0001298. doi: 10.1371/journal.pgph.0001298 (PMC10022376; doi:10.1371/journal.pgph.0001298)
Supplement: Attachment — Submitted filename: 08.09.2022 Point by point responses to reviewers comments.docx [file pgph.0001298.s002.docx]

Reviewer #1:

Your paper presents an important work.

General comment: You cannot incorporate a new finding in the discussion part that has never existed in the result section. Discussion section should be a condensed & summarized form of the results section, NOT vice versa! Again better you strengthen your discussion by discussing with similar studies conducted elsewhere and justify some inconsistencies in your study as compared with others.

Response: We have revised the manuscript as the reviewer suggested. In addition, we have compared and contrasted our findings with those of other studies in the context of a similar socioeconomic setting.

Line number 47: Change to: ….were assessed for structural quality of ANC …Line number 69-70: I didn’t see training is a problem in the result section above. Line 102: Check the bracket for references. Line 136: citation. Line 139: ….on the status …
Line 160: change was to were.

Response: We corrected all of these errors in the revised version.

Line 194-195: How did you dichotomize (Poor Better)? Do you have any reference? If so, would you cite it?

Response: We corrected in the revised version and cited them using previous studies that used mean as cut off point for dichotomisation of quality score: poor (if score less than mean) and optimal (score equal or more than mean).

Line 194-195: why did you select ANC first as quality indicator? Why not ANC 4? Do you have a reason?

Response: We have corrected this for ANC services in general rather than only first ANC visit.

Line 258 :( 76/9 %????) Make it clear. Line 271-272: what it mean by low availability (write the actual figure.). Table 2: Why you over looked the general readiness assessment result. eg. Mobile phone. Again in the era of Covid 19, how you overlooked about the availability of alcohol for hand rub?

Response: We thank the reviewer for pointing this for the correction. We have corrected this, including the availability of mobile and landline phones and commodities such as alcohol for hand rubs.

Table 4: Check N for provinces. Check for the order of the tables

Response: We thank the reviewer for pointing out this editorial comment. We have corrected the revised version, including the order of the tables.

On discussion part, in the first paragraph the last two sentences look recommendations. Why?

Response: We agreed with the reviewer. We have incorporated those statement as recommendations in the subsequent paragraph of the discussion.

Again, why you overlooked the general assessment result both in discussion and conclusion section?

Response: We have corrected the status of general readiness in all sections and revised it accordingly.

Reviewer #2:

This manuscript focuses on an important subject regarding the availability and quality of service provided to women and newborns in health facilities in Nepal providing antenatal care, delivery and postnatal care. The paper is technically sound and coherent, and the results support the conclusion. However, the source of data is a national survey conducted in 2015. While the use of secondary data for research can be very useful in providing good insights as they often have a wide sample size that is representative of the country in focus, but they often report data from a wide time period (usually 4-5 years). In addition, the data source can sometimes be outdated if a newer survey has been conducted. I conducted an online review and observed that there is a more recent preliminary survey report Nepal Health Facility Survey (NHFS) for 2021 here <https://nepalhealthmag.com/nepal-health-facility-survey-2021-preliminary-report/#:~:text=The%202021%20Nepal%20Health%20Facility%20Survey%20%28NHFS%29%20is,those%20services%2C%20and%20the%20quality%20of%20client%20services>. This may have been released after the manuscript was submitted and may show a different picture of the state of structural readiness in these facilities. Based on the above, I recommend that the paper be published with minor changes to the title showing the time period E.g., Health system readiness (structural quality) of HFs for routine maternal and newborn health services in Nepal: Evidence from nationally representative health facility survey conducted between 2009 and 2014 (Adjusted to show time frame of survey report). Alternatively, the dataset used to arrive at the conclusions in the paper can be updated to more recent report data.

Response: We thanked the reviewer for this feedback. Our analysis was based on the latest facility survey data derived from the Nepal Health Facility Survey 2015. We adopted the Service Availability and Readiness Assessment (SARA) framework to assess the structural quality of health facilities for antenatal and perinatal services.

We agree that Nepal has released a preliminary report of the second Nepal Health Facility Survey 2021. However, we expect data of NHFS 2021 will release and available by mid of 2023 for further analysis. The first author analysed the available NHFS 2015 dataset for his doctoral research. This manuscript is based on the first author’s doctoral thesis, which was approved by the University of Queensland, Australia. In this context and with the availability of data on health facility surveys, the findings of this study will be the baseline for the further analysis of upcoming NHFS data and similar analysis of other health facility surveys in other countries.

As per the reviewer's suggestion, we have indicated the year of the health facility survey in the title. Unlike the household survey (e.g., Nepal Demographic and Health Surveys which collects information five years preceding the survey), the health facility survey collects information on the day of the survey. For instance, NDHS 2016 collected information from women five-years preceding the survey (e.g., 2011-2016), and NHFS 2015 collected information during the survey rather than previous information like NDHS.

Reviewer #3:

I congratulate the authors for assessing the availability and protection of services in maternal and neonatal health care. However, the results indicate that private health units have quality structures to offer better care to mothers and newborns than public health units in remote communities, which I think is to be expected. It would be important for this evaluation to be carried out among the public sector health units.

Response: Yes, quality of care was better/optimal in private facilities and poor in public facilities. As most pregnant women’s ANC visits and delivery occur in Nepal's public facilities, the quality of care should be improved in the public facilities. Therefore, we have revised the discussion section focusing on strategies to improve the quality of care in public facilities.

Reviewer #4:

The manuscript title: “Health system readiness (structural quality) of HFs for routine maternal and newborn health services in Nepal: Evidence from nationally representative health facility survey.” This manuscript seems scientifically well written. The study used nationally representative data collected in 2015. However, the claim made by the manuscript is relevant to the present context of Nepal. After the federal system, local governments are establishing community-level health facilities, and the recommendations made by the manuscript will be applicable for maintaining quality MNH service.

Response: we thank you for the feedback. We agree with the reviewer that in the changing context of the federalised health system, there is a good opportunity to strengthen the local health system for better quality health services, including maternal and newborn health services.

The manuscript seems fit for the journal; however, I do have some minor comments in the following sections: Please insert line number in your text (there is no line number after page 13)

Response: we have inserted line numbers in the remaining pages as suggested

Methods/study variables:1) Line 189: All the independent variables are self-explanatory; however, I think it is better to explain bit or refer about mechanism of quality assurance (independent variables).

Response: we have revised the quality assurance mechanisms referring the questionnaire of NHFS 2015. The NHFS questionnaire referred quality assurance mechanisms as availability of a report or minutes of a quality assurance meeting, a supervisory checklist, a mortality review, or an audit of records or registers

2) Line 193 to 195: “This study has two outcome variables: Structural quality of HFs for i) first ANC visit 194 (poor, better), ii) delivery and postnatal services (poor, better). Structural quality of 195 HFs for the first ANC visit, and delivery and PNC services”. I think we can minimize duplication.

Response: We used data derived from the NHFS 2015. We have taken two outcome variables of interest in our study. The quality of care was assessed in 901 health facilities (HFs) accredited for ANC services. Similarly,454 HFs were accredited for the delivery of childbirth services. In Nepal, all HFs that provide perinatal (delivery and postnatal services) services provide ANC services but those facilities that provide ANC services might not be accreted to provide childbirth services. This applies to our sample too. Future research can be conducted by taking a sample of health facilities provide both antenatal and perinatal services. We have recommended as a future research implication.

Results: 3) Line 258: “More than three in four (76/9%)”. I think it is 90% (Table 1)

Response: we have corrected it as suggested.

4) Table 1:- I think author need to write abbreviation in the legend for the readability

Response: we have corrected it as suggested.

5) Page 14: “Distribution HFs with better structural quality for MNH services” -“of” is missing.

Response: we have corrected as suggested.

6) Page 14 second paragraph: “On the other hand, the HFs of the Mountain region and Karnali province (12%) had poor structural quality for delivery and postnatal services compared to province one (38%)”- I think it is better to remove the "mountain region" as it is not study variable (for this manuscript).

Response: we have corrected it as suggested.

7)For table 4 and 3 - Re-arrange the table number (4 came before 3). Please make consistency in upper/lower case while writing abbreviations in legend.QA: Quality assurance; HF: health facility, PHCCs: primary health care centers; HPs: health posts; ANC: Antenatal care; PNC: postnatal care

Response: we have corrected it as suggested.

Reviewer #5:

I have well read your work which i found very interesting and original. However, i have some questions: 1) in the methods section, you said WHO's SARA manual was the one you used to assess the structural quality of health facilities to provide ANC and delivery and post-natal services. However, i saw for ANC services, 21 items that you used whereas in the manual, there are only 06 items. The same observation has been made for the delivery and post-natal services. I understand that you have contextualized the items since you added and removed some items. Please could you provide clear references and deep explanation on the choice of items you added to the list and those you removed from SARA's items?

Response: We thanked the reviewer for the feedback. We agree with the reviewer that we took SARA as the guiding framework of analysis. We contextualised the structural quality of HFs assessment based on available information in the NHFS 2015. Referring to the Nepals’ national guideline for maternal and neonatal care and other relevant studies in Nepal, we have taken the list of items to assess the structural quality of the health facility for MNH services.

2) As for the readiness, SARA's manual has presented 04 domains (staff and guidelines, equipment, diagnostic and medicines and commodities). I have understood that you added general readiness as domain and did not include the diagnostic domain. Could you clearly explain that?

Response: As described in the methods section, HF's structural quality of HFs includes domains (service availability and facility readiness). The first domain talks about the availability of technical services (not necessary to have services on the day of the health facility survey. For instance, if there is the provision of ANC services in the HFs, HFs should be ready if pregnant women come there for ANC services). At the same time, facility readiness component covers general readiness, medicine, staff and guideline, equipment and commodities. We have assessed the structural quality of HFs for MNH services accordingly. We have detailed those procedures in the methods section.

3) in the results, the way you presented your tables especially tables 2 and 3 is not easily understandable by readers. Please, could you present the tables consistency?

Response: we have presented tables. We have described it narratively.

4) you have calculated scores to define the structural quality of health facilities using availability score and readiness score for each service. Based on the SARA manual, the way to calculate scores depends on the domains and items in each domain. Please, could you clearly explain the way you calculated facility readiness score based on the domains? Also, try to explain it in your methods section.

Response: We have mentioned details in the method section and a list of items included in the annexe. Structural quality scores were based on the availability of domains/subdomains specific items presented in Tables 2 and 3 (where presented in %, and aggregate scores are presented in decimal points (out of 1.00)). As described in response to comment two above, we have covered subdomain-specific structural quality and then averaged them to get the final score, which we have presented in Figures 1 and 2.
